# Supplementary material for: Using Machine Learning to Discover Latent Social Phenotypes in Free-Ranging Macaques
Source: Brain Sci. 2017 Jul 21;7(7):91. doi: 10.3390/brainsci7070091 (PMC5532604; doi:10.3390/brainsci7070091)
Supplement: Supplementary file 1 [file brainsci-07-00091-s001.pdf]

Supplementary Material

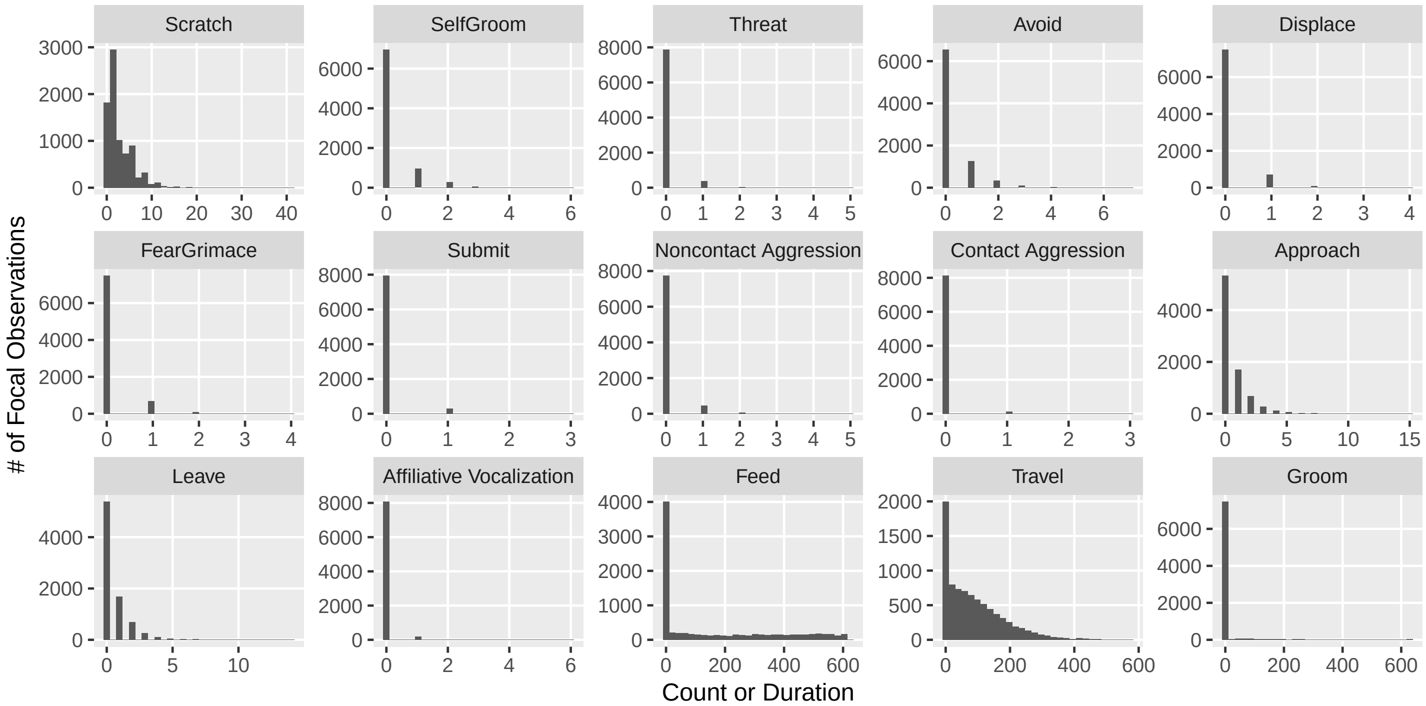

Figure S1: Distribution of behavior amounts across focal observations. Note that behaviors are not separated into give/receive as they are in the model, so the fit data has a higher proportion of zeros per behavior than presented here.

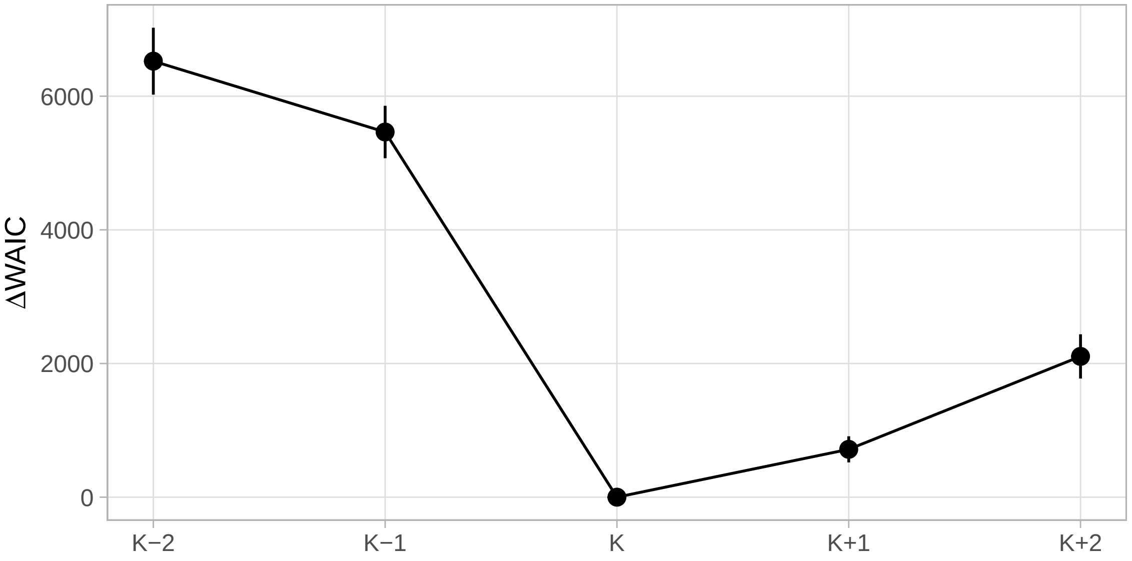

Figure S2: Choosing number of states for simulated data using WAIC. Y-axis shows difference in WAIC between the model with the correct number of states, K, and models with more and fewer states. Error bars represent two standard errors. Lower is better.

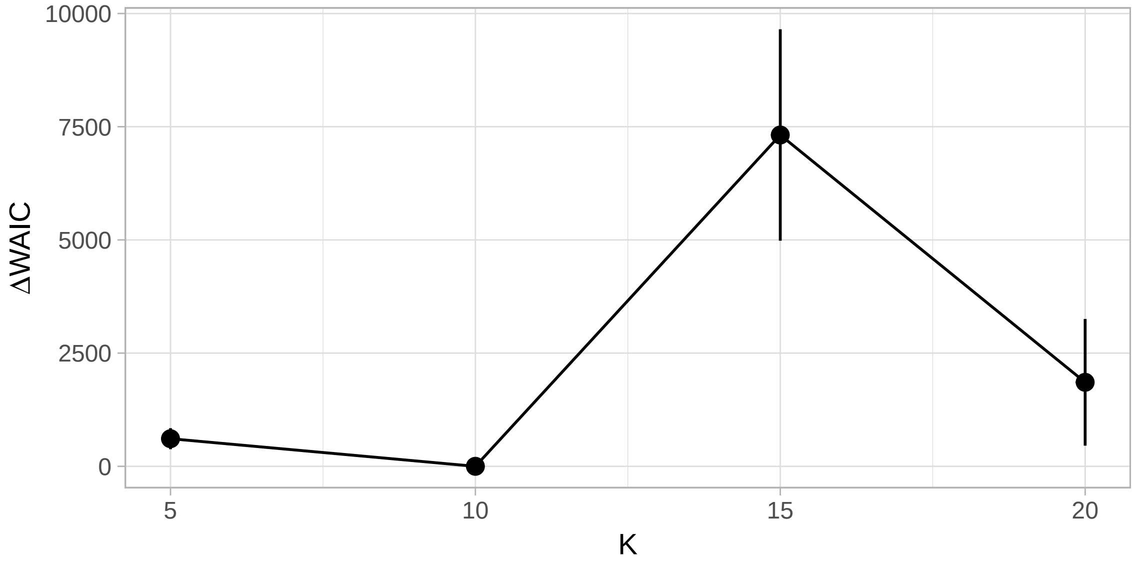

Figure S3: Choosing number of states for the Cayo Santiago data using WAIC. Y-axis shows difference in WAIC between the model with 10 states and models with more and fewer states. Error bars represent two standard errors. Lower is better.

	Factor1	Factor2	Factor3	Factor4	Factor5	Factor6	Factor7	Factor8	Factor9	Factor10
Scratch	−0.034	−0.023	−0.028	−0.014	0.031	0.7	...	0.019	−0.037	−0.01
SelfGroom	−0.018	0.013	...	...	0.015	0.39	−0.079	−0.14	0.013	...
Threat(give)	...	...	0.03	...	...	−0.022	0.12	...	−0.032	0.17
Threat(receive)	...	0.018	...	0.019	...	...	...	0.048	...	0.25
Avoid(give)	...	0.011	0.02	0.014	−0.056	−0.063	0.44	...	−0.016	0.077
Avoid(receive)	0.012	0.047	0.02	0.032	−0.063	−0.066	−0.043	0.37	0.12	0.16
Displacement(give)	0.014	0.015	−0.014	...	−0.029	−0.011	0.31	0.05	0.029	−0.032
Displacement(receive)	−0.026	0.012	−0.016	...	−0.021	−0.03	...	0.17	0.4	0.097
FearGrimace(give)	...	...	0.013	−0.023	0.045	0.017	−0.042	0.086	0.12	0.16
FearGrimace(receive)	−0.03	...	...	...	0.04	0.027	0.39	−0.082	...	0.014
Submit(give)	0.018	0.018	0.046	0.044	−0.013	−0.052	−0.087	0.16	0.027	0.12
Submit(receive)	0.015	...	...	...	...	−0.02	0.22	...	...	0.023
NonContactAgg(give)	0.017	−0.012	0.024	...	−0.025	0.015	0.15	...	...	0.32
NonContactAgg(receive)	...	−0.025	...	−0.039	−0.014	...	0.039	0.064	...	0.36
ContactAggression(give)	...	0.017	0.029	0.029	0.015	−0.011	0.095	0.013	0.018	0.08
ContactAggression(receive)	−0.012	0.028	−0.03	0.062	0.014	...	−0.027	...	0.069	0.16
Approach(give)	0.21	0.41	−0.053	0.68	0.12	...	0.12	0.11	0.1	0.11
Approach(receive)	0.21	0.23	0.9	0.14	0.18	−0.03	0.069	0.1	0.12	0.023
Leave(displace)	0.077	...	0.079	0.053	0.053	...	0.048	−0.03	0.52	−0.018
Leave(give)	0.19	0.91	0.22	0.17	0.11	−0.02	0.12	0.13	0.02	0.055
Leave(receive)	0.25	−0.041	0.42	0.67	0.15	−0.029	0.042	0.11	0.025	0.071
AffilVocal(give)	0.016	...	0.018	...	0.09	0.034	0.018	...	0.026	...
AffilVocal(receive)	0.018	...	...	...	0.052	0.031	...	0.012	...	...
Groom(give)	...	0.07	−0.038	0.096	0.42	−0.024	−0.045	−0.067	...	...
Groom(receive)	...	0.015	0.067	0.042	0.47	−0.012	−0.032	−0.046	...	−0.019
Feed	−0.011	0.036	0.072	0.065	−0.35	−0.29	0.19	0.46	0.063	0.029
Travel	−0.12	0.069	...	0.047	−0.17	0.079	0.34	0.34	...	0.097
PassiveContact	0.097	0.023	...	...	0.3	−0.066	−0.017	−0.057	−0.032	...
SocialProximity	0.89	0.087	0.094	0.12	0.19	−0.032	−0.01	−0.04	0.029	...
ProximityGroupSize	0.95	0.11	0.12	0.15	0.18	−0.025	0.021	0.022	0.054	...

Table S1. Factor loadings for factor model 1. Loadings below 0.01 are ommitted for clarity.

	Factor1	Factor2	Factor3	Factor4	Factor5	Factor6	Factor7	Factor8	Factor9	Factor10
Scratch	−0.29	−0.039	−0.02	0.17	−0.073	0.11	0.48	−0.038	−0.054	−0.031
SelfGroom	−0.16	...	−0.077	0.23	...	−0.015	0.12	−0.095	...	...
Threat(give)	...	0.052	0.22	...	0.095	0.13	0.18	0.084	0.19	0.11
Threat(receive)	0.012	0.48	0.036	0.028	...	0.045	0.15	...	0.035	...
Avoid(give)	...	−0.23	0.76	0.032	0.026	0.011	−0.047	0.055	−0.027	−0.024
Avoid(receive)	0.04	0.73	−0.11	−0.15	0.067	−0.16	−0.31	0.063	0.077	−0.077
Displacement(give)	−0.026	−0.083	0.4	−0.014	0.059	0.096	0.022	−0.11	0.052	0.011
Displacement(receive)	0.05	0.59	−0.074	−0.15	0.38	−0.011	−0.068	0.056	...	0.21
FearGrimace(give)	0.06	0.31	−0.091	0.12	0.17	−0.047	−0.2	−0.01	0.02	...
FearGrimace(receive)	−0.033	−0.27	0.82	0.17	−0.074	0.03	−0.034	0.16	0.039	−0.01
Submit(give)	0.19	0.44	−0.15	−0.12	0.024	−0.072	−0.051	0.033	−0.057	0.063
Submit(receive)	0.11	0.096	0.46	−0.031	...	−0.06	0.083	0.016	...	0.012
NonContactAgg(give)	...	−0.031	0.28	−0.13	−0.037	0.38	0.45	−0.06	0.059	−0.018
NonContactAgg(receive)	−0.093	0.34	−0.092	−0.091	−0.32	0.32	0.07	−0.16	−0.052	−0.094
ContactAggression(give)	0.028	...	0.038	0.068	0.026	0.51	0.099	0.045	0.037	0.013
ContactAggression(receive)	0.039	0.25	−0.1	0.076	...	0.15	0.13	0.039	0.05	0.029
Approach(give)	0.64	0.24	0.21	0.16	0.12	0.033	−0.039	0.19	0.63	0.046
Approach(receive)	0.67	0.21	...	0.12	0.18	0.17	−0.22	0.34	...	0.36
Leave(displace)	0.23	0.27	0.066	−0.041	0.65	0.044	−0.036	0.01	0.056	−0.02
Leave(give)	0.66	0.18	0.11	0.09	0.11	0.013	−0.077	0.66	0.24	0.029
Leave(receive)	0.73	0.32	0.076	0.11	0.027	...	0.01	0.012	0.28	0.52
AffilVocal(give)	0.18	−0.028	−0.15	0.13	...	0.16	0.069	−0.027	−0.02	...
AffilVocal(receive)	0.21	−0.12	−0.21	0.038	0.02	0.08	−0.12	0.012	0.17	...
Groom(give)	0.31	0.15	...	0.55	−0.076	0.085	−0.077	0.15	0.15	...
Groom(receive)	0.26	−0.11	−0.028	0.55	0.026	0.12	...	0.14	0.091	0.11
Feed	...	0.13	−0.16	−0.62	0.015	−0.038	−0.065	0.031	0.056	0.052
Travel	−0.26	−0.3	0.31	−0.41	−0.21	0.14	0.16	0.17	0.11	...
PassiveContact	0.32	−0.17	0.08	0.31	−0.078	0.37	−0.088	−0.063	−0.011	0.04
SocialProximity	0.98	0.042	...	0.13	0.11	−0.025	−0.03	...	−0.028	−0.069
ProximityGroupSize	0.95	0.097	−0.032	0.078	0.12	0.056	−0.12	...	0.018	−0.049

Table S2. Factor loadings for factor model 2. Loadings below 0.01 are ommitted for clarity.
